# Numerical Analysis of the Forming Mechanism of Exit Burrs in Metal Milling under Ice Boundary Constraint

**DOI:** 10.3390/ma15165546

**Published:** 2022-08-12

**Authors:** Chengxin Wang, Wentao Xiong, Guo Ding, Pengchao Li, Zhixiang Zhu, Haibo Liu

**Affiliations:** 1 Key Laboratory for Precision and Non-Traditional Machining Technology of Ministry of Education, Dalian University of Technology, Dalian 116024, China; 2 Measuring and Testing Institute under Xi′an Aerospace Corporation, Xi′an 710100, China

**Keywords:** exit burrs, ice boundary constraint (IBC), forming mechanism, finite element model (FEM), burr height

## Abstract

In metal processing, exit burrs are usually inevitable, which is a huge challenge for high-precision manufacturing. This paper innovatively proposes an ice boundary constraint (IBC) method to actively suppress exit burrs to obtain better workpiece edge quality. Firstly, the formation mechanism of the exits burr is analyzed from the perspective of material flow at the edge of the workpiece, and the principle of the IBC method is explained. Secondly, a finite element model (FEM) is established to analyze the stress distribution and flow at the edge of the workpiece, so as to reveal the suppression mechanism of IBC on the exit burrs. Finally, the feasibility of IBC method and the validity of FEM are verified by the milling experiments. The experimental results show that the IBC method can reduce the exit burr height by 51.4% on average, and FEM can effectively predict the height of the exit burr. The IBC method proposed in this study can provide some reference and guidance for the active suppression of exit burrs in industry.

## 1. Introduction

In the process of metal milling, the edge of workpiece inevitably produces burrs, which has always been the biggest obstacle to high-precision machining and automated production. The formation of burr will reduce the quality of the workpiece edge and the integrity of the machined surface [1], thereby reducing assembly accuracy and service performance. In addition, during the service process of the parts, the contact load between the assembly surfaces will cause the burrs to fall off and accumulate in the equipment, thus increasing the probability of equipment failure [2]. To remove the burr, the craftsmen add a deburring process after the part is manufactured [3,4]. However, the deburring process can increase manufacturing costs. Therefore, the realization of burr-free processing of products has always been the focus of scholars’ research.

Studying the formation process of burrs is a necessary premise to control and minimize burrs, and it has certain guiding significance for exploring active burr suppression methods. The analytical method is currently the main method for people to analyze the burr formation mechanism and explore the relationship between cutting variables and burr geometry [5]. Zhang et al. [6] investigated the burr formation mechanism in micro end milling by considering the minimum chip thickness, as well as the size effect of cutting edge radius. Additionally, they proposed an improved analytical model to predict the size of Poisson burrs and exit burrs, and they carried out milling experiments to verify the accuracy of the model. Pang et al. [7] investigated material lateral flow and Poisson burr formation mechanism, and developed an improved analytical model based on mechanical, geometric and material properties to describe surface and subsurface plastic deformation and Poisson burr morphology. Then, experimental verification showed that the proposed analytical model can effectively predict the height and thickness of the Poisson burrs. Hassan et al. [8] developed a new analytical model to describe entrance burr formation with tool geometry, operating parameters and workpiece material properties as variables.

Besides the analytical method, the finite element method is also the main method to analyze the stress–strain distribution and plastic deformation behavior during burr formation. Luan et al. [9] established a finite element model based on the Johnson-Cook constitutive model to analyze the formation mechanism of the top burr and predict the height of the top burr. Then, milling experiments were performed to verify the simulation results. Olsson et al. [10] studied the effects of variable depth of cut, feed and main cutting edge angle on burr formation in oxygen-free copper machining through finite element simulation. By comparing the simulation results with the experimental results, it can be seen that the reduction in the depth of cut and the main cutting edge angle results in a reduction in burr formation. Yadav et al. [11] established a finite element model of the side exit burr on the upward milling based on the Johnson Cook material constitutive model, and predicted the height and width of the burr. Then, it was found that the burr height and width obtained from the simulation has been verified experimentally with a maximum error of 15%.

On the basis of analyzing the mechanism and influencing factors of burr formation, it is also crucial to find a suitable active burr suppression method. At present, common active burr suppression methods include chamfering, optimizing cutting parameters, changing tool geometry, etc. [12,13]. Wu et al. [14] established a new burr-free theoretical model based on the analysis of burr formation, and pointed out that changing the exit angle of the workpiece is the key to realizing burr-free interrupted cutting. Gaitonde et al. [15] used Taguchi’s quality loss function method to determine the optimal combination of cutting speed, feed, point angle and lip clearance angle for specified drill diameters, so as to minimize burr height and burr thickness during drilling of AISI 316L stainless steel workpieces.

There are many factors affecting burr formation, such as temperature field, tool wear [16] and so on. Akula et al. [17] found that the reduction in the processing temperature can significantly reduce the burr size during helical milling of titanium alloys. Kizhakken et al. [18] proposed a mathematical model to predict burr thickness during micro-end milling of Ti6Al4V. It can be seen from the proposed model that temperature is a key factor affecting the size of the burr. Asad et al. [19] proposed to optimize the tool geometry in the machining process to reduce tool wear, so as to reduce the formation of burrs.

This paper proposes an IBC method to suppress exit burrs. Firstly, the formation mechanism of the exit burr is explained from the perspective of material flow, and the working principle of the IBC method is described. Then, a finite element model is established to analyze the stress distribution and flow state, so as to reveal the suppression mechanism of the IBC on the exit burr. Finally, the milling experiments are carried out to verify the effectiveness of the IBC method and the prediction accuracy of the FEM. Research in this paper provides a valuable reference for realizing active suppression of exit burrs.

## 2. Analysis of the Formation Process of Exit Burr

**Figure 1:**
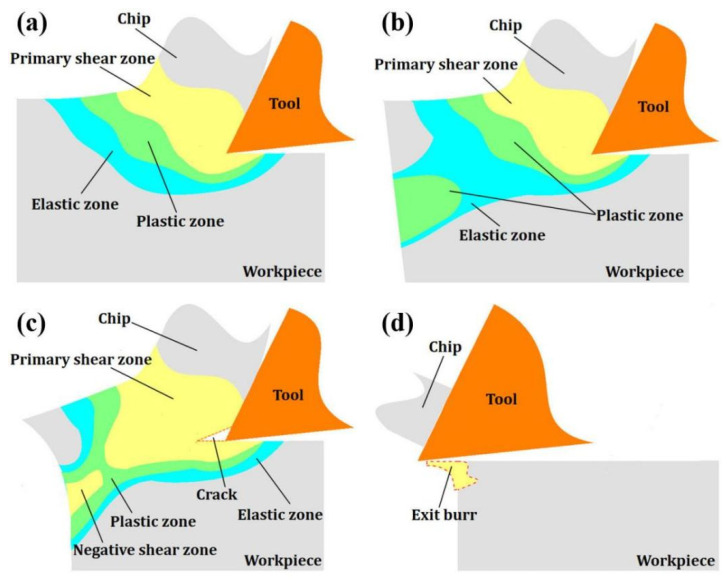
The formation process of the exit burr: (**a**) The stress distribution around tool tip; (**b**) The changes in stress distribution state; (**c**) The generation of crack and negative shear zone; (**d**) The formation of exit burr.

During machining, the primary shear zone, plastic zone and elastic zone of the workpiece material are formed by the extrusion and shearing of the cutting edge (Figure 1a). As the tool moves towards the edge of the workpiece, the elastic zone and the plastic zone reach the edge of the workpiece, respectively. At the same time, bending deformation occurs at the workpiece edge. The plastic deformation then extends to mix with other plastic deformation zones at the workpiece boundary (Figure 1b). Then, as the cutting progresses, crack will be created in the primary shear zone, and continue to extend along the cutting line due to the shear force. At the same time, a negative shear zone is generated at the material edge (Figure 1c). The crack separates the chip along the cutting line, and the exit burr is left on the edge of machined workpiece surface (Figure 1d).

## 3. The Principle of the Ice Boundary Constraint (IBC) Method

**Figure 2:**
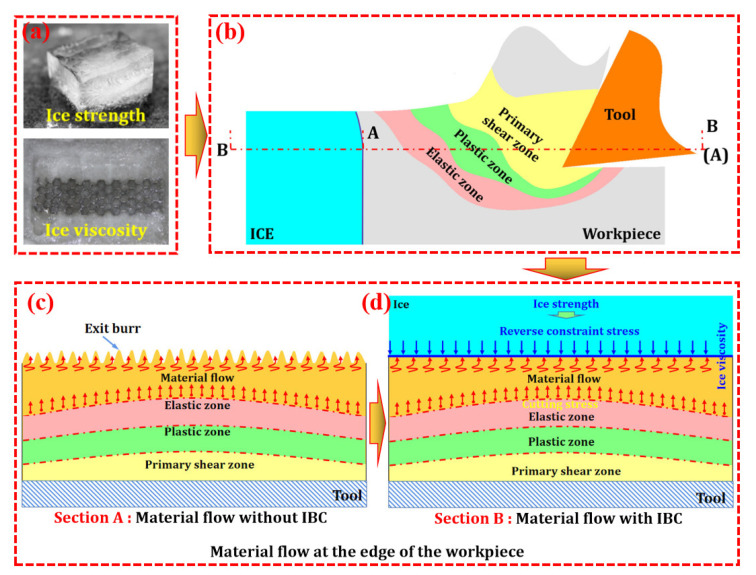
The principle of IBC method: (**a**) The strength and viscosity of ice; (**b**) Schematic diagram of IBC (A represents section A; B represents section B); (**c**) Material flow at the edge of the workpiece; (**d**) Material flow at the edge of the workpiece under IBC.

Ice is a crystal formed by the orderly arrangement of water molecules, which are linked together by hydrogen bonds to form a very “open” (low density) rigid structure, and has natural properties such as viscosity and rigidity. Applying these natural properties of ice to industrial production is the key to achieving clean processing. In this paper, an IBC method is proposed to suppress exit burrs at workpiece edge. IBC uses the fluidity of water and the viscosity and strength of ice to constrain the edge of the workpiece (Figure 2a) and improve the quality of edge milling. The fluidity allows the water to adapt to the various surface shapes of the workpiece and fill the structural space of the workpiece, thereby ensuring that the ice adheres closely to the surface of the workpiece after the water freezes. Additionally, ice itself is not viscous. The so-called viscosity of ice essentially means that the ice directly contacts the surface of the object, so that the water on the surface of the object freezes rapidly, thereby gluing the ice and the object together. The strength of ice refers to the ability of ice to resist damage under the action of external force.

During machining, the cutting stress created by the tool drives the workpiece material towards the edge (Figure 2b), and as the tool approaches the workpiece edge, the edge material continuously flows to the ice–workpiece interface (Figure 2c), which creates extrusion stress on the ice. Since the action of force is mutual, this extrusion stress can be considered as the reverse constraint stress of ice on the edge material, and the maximum value of the reverse constraint stress can be considered as unidirectional compressive strength of ice. This reverse constraint stress can suppress the material flow and elastic and plastic bending of the workpiece edge caused by the extrusion stress (Figure 2d), thereby improving the quality of the workpiece edge and the integrity of the machined surface.

## 4. Numerical Analysis

### 4.1. Material Constitutive Model

AA2024 is widely used in advanced equipment in aerospace, automobile, ship building and other fields. During machining, plastic deformation of AA2024 can lead to the generation of exit burrs, which reduce edge quality and surface integrity. Therefore, AA2024 is selected as the workpiece material in this paper. The physical properties of workpiece, tool and ice are listed in Table 1. The geometric parameters of the tool are listed in Table 2.

The constitutive model of the material is essential to accurately simulate the cutting state and stress–strain distribution. The Johnson–Cook (J-C) constitutive model can well describe the ultimate strength and failure process of metal materials under large strain and high strain rate. Thus, the J-C model is utilized as the constitutive model of AA2024, which can be expressed as:

(1) $$\sigma =\left(A+B\varepsilon^{n}\right)\left[1+Cln\left(\frac{\dot{\varepsilon }}{{\dot{\varepsilon }}_{0}}\right)\right]\left[1-{\left(\frac{T-T_{r}}{T_{m}-T_{r}}\right)}^{m}\right]$$

where σ is the flow stress, ε is the plastic strain, $\dot{\varepsilon }$ is the plastic strain rate, $\dot{\varepsilon_{0}}$ is the equivalent plastic strain, T is the material temperature, $T_{r}$ is the room temperature, $T_{m}$ is the melting temperature, and A, B, C, n and m are J-C constitutive constants for AA2024 (Table 3).

To describe the damage characteristic of AA2024 during cutting, J-C damage model was adopted as:

(2) $${\bar{\varepsilon }}_{f}^{pl}=\left[D_{1}+D_{2}\left(D_{3}\frac{p}{q}\right)\right]\left[1+D_{4}ln\left(\frac{\dot{\varepsilon }}{{\dot{\varepsilon }}_{0}}\right)\right]\left[1-D_{5}{\left(\frac{T-T_{r}}{T_{m}-T_{r}}\right)}^{m}\right]\;$$

(3) $$w=\sum \frac{\Delta {\bar{\varepsilon }}^{pl}}{{\bar{\varepsilon }}_{f}^{pl}}$$

where $\Delta {\bar{\varepsilon }}^{pl}$ is the increment of the equivalent plastic strain, and $\Delta {\bar{\varepsilon }}_{f}^{pl}$ is the equivalent strain of fracture. When “w=1”, the workpiece material begins to fracture. $D_{1}$, $D_{2}$, $D_{3}$, $D_{4}$ and $D_{5}$ are J-C damage constants for AA2024 (Table 4)

In order to accurately describe the stress–strain state of ice during processing, this paper adopts the Tsai–Wu criterion [20] as the yield criterion of ice, which can be expressed as

(4) $$f\left(\sigma_{m},q\right)=\frac{3}{2}q^{2}-\left(a_{0}+a_{1}p+a_{2}p^{2}\right)$$

where $a_{0}$, $a_{1}$ and $a_{2}$ are the constant values (Table 5), p is hydrostatic stress (p = −$\sigma_{m}$), and q is Octahedral shear stress.

According to Drucker’s assumption, the constitutive equation of ice is established by using the strain increment theory, which can be expressed as:

(5) $$\varepsilon =\int \frac{1}{2G}d\sigma_{ij}-\int \frac{3\mu_{i}p}{E}d\delta_{ij}+\int \frac{\partial f^{T}}{\partial \sigma_{ij}}d\lambda_{i}$$

where $d\sigma_{ij}$ is stress increment, $\delta_{ij}$ is the Kronecker symbol, and $\mu_{i}$ and $\lambda_{i}$ are Lame coefficient.

To describe the failure behavior of ice during cutting, the failure criterion based on equivalent plastic strain $\varepsilon_{eq}^{p}$ and hydrostatic stress p is adopted, which can be calculated as:

(6) $$\varepsilon_{eq}^{p}=\sqrt{\frac{2}{3}\varepsilon_{ij}^{p}\varepsilon_{ij}^{p}}$$

(7) $$\varepsilon_{f}=\varepsilon_{0}+{\left(\frac{p}{10^{8}}-0.5\right)}^{2}$$

where $\varepsilon_{eq}^{p}$ is the equivalent plastic strain, $\varepsilon_{f}$ is the failure strain, $\varepsilon_{0}$ is the initial failure strain, and p is the hydrostatic pressure. In the failure criterion, $\varepsilon_{0}$ is the input parameter. If $\varepsilon_{eq}^{p}>\varepsilon_{f}$, the unit will be invalidated and deleted.

### 4.2. Friction Coefficient

In the actual cutting process, friction has a certain influence on the formation of exit burrs. Therefore, in order to better reflect the formation process of the exit burr, the friction needs to be fully considered in the finite element model. According to Coulomb’s friction law, the friction model is expressed as:

(8) $$\tau_{f}\leq \mu \sigma_{n}$$

where μ is the friction coefficient, $\tau_{f}$ is the friction between the tool and the machined surface, and $\sigma_{n}$ is the normal stress. In this paper, the friction coefficient μ is 0.2.

### 4.3. Cryogenic Cooling Environment

Cutting heat and ambient heat can weaken the constraint effect of ice on the edge of the workpiece, so cooling is an essential link during the cutting process. In actual processing, liquid nitrogen (LN2) is sprayed into the machining zone to prevent the ice from melting. Thus, the heat transfer mode can be set to forced convection heat transfer in the finite element model. The heat dissipation density $q_{h}$ can be expressed as:

(9) $$q_{h}=\bar{h}\left(T_{f}-T_{r}\right)$$

where $T_{f}$ is the tool temperature, and $T_{r}$ is the room temperature. $\bar{h}$ is the average convective heat transfer coefficient, and it can be obtained as:

(10) $$\bar{h}=\frac{0.664P_{r}^{1/3}R_{e}^{1/2}K_{air}}{L_{e}}$$

where $L_{e}$ is the effective cooling strength, $K_{air}$ is the heat transfer coefficient of air, $P_{r}$ is the Prandtl number, and $R_{e}$ is the Reynolds number. $P_{r}$ and $R_{e}$ are determined by the properties of LN2. Due to the plastic deformation of the workpiece material, the friction between the tool and the chip, and the friction between the tool and the workpiece, a large amount of cutting heat will be generated during the cutting process, so that LN2 sprayed into the machining area is vaporized, thereby improving the heat transfer efficiency. Therefore, the actual heat transfer coefficient ${\bar{h}}_{e}$ can be calculated as:

(11) $${\bar{h}}_{e}=\lambda \bar{h}$$

where λ is the compensation coefficient for the heat exchange between LN2 and the surrounding environment and the vaporization of LN2. Therefore, in order to accurately simulate the cooling environment, the value of ${\bar{h}}_{e}$ is 20 W/m·K in simulation.

### 4.4. Cutting Simulation

**Figure 3:**
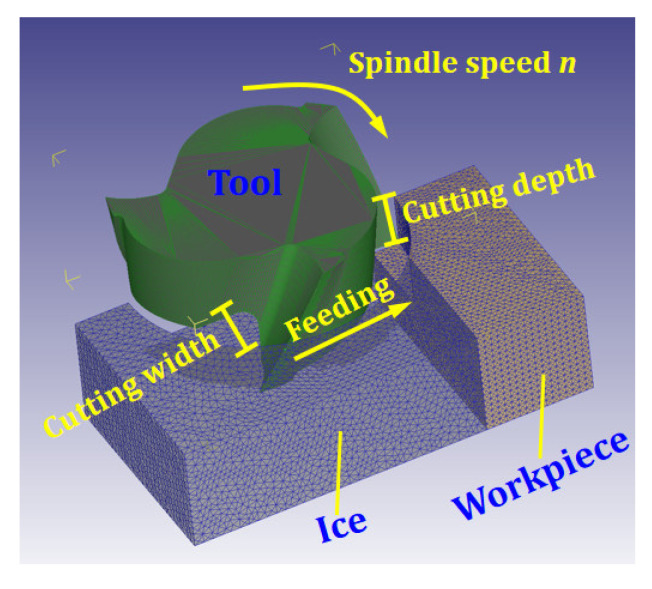
Milling simulation of workpiece under IBC.

In the simulation, a volume of ice is placed on the edge of the workpiece to suppress exit burrs (Figure 3). The boundary conditions are listed in Table 6. The size of the workpiece is 8 mm × 4 mm × 4 mm, the size of the ice is 8 mm × 11 mm × 4 mm, and the initial temperature of ice is −20 °C. The tool used is a three-blade milling cutter, made of tungsten steel carbide. The radial depths of cut are 1 mm and 2 mm. The axial depth of cut is 2 mm. The spindle speed is 4000 r/min and 5000 r/min.

### 4.5. Simulation Results Analysis

**Figure 4:**
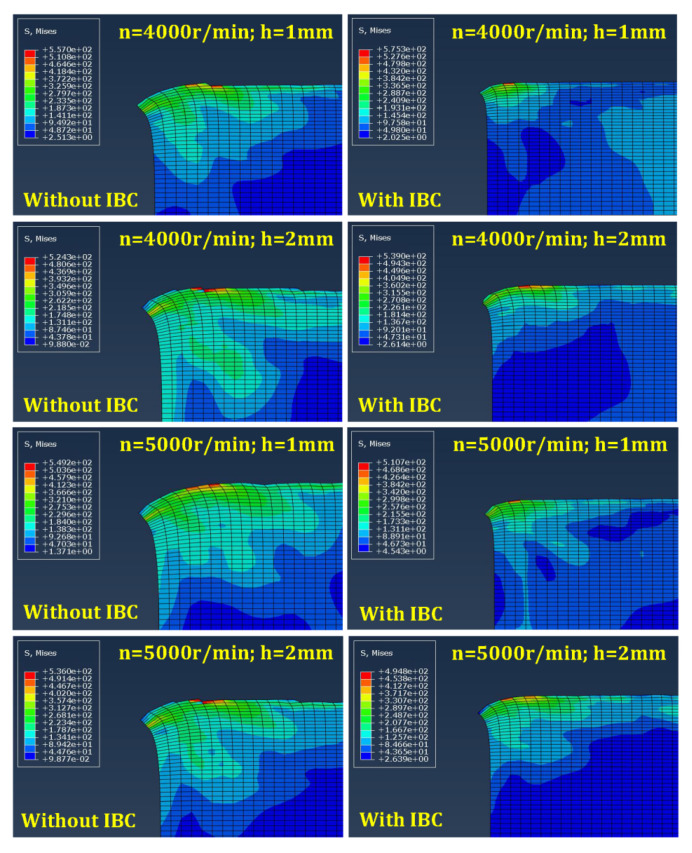
Simulation results of exit burrs height.

According to the simulation results (Figure 4), it can be seen that the IBC method can effectively suppress the formation of exit burrs under different cutting parameters, thereby improving the quality of the workpiece edge and the integrity of the machined surface. In addition, the suppression effect of the IBC method on the exit burrs is less affected by the variation in cutting parameters.

During the machining process, the material flow at the edge of the workpiece is constantly flowing, which is mainly determined by the cutting stress and plays a decisive role in the formation of the exit burr. To analyze the formation process of the exit burr, the stress flow and distribution at the workpiece edge are simulated by FEM. Taking *n* = 4000 r/min, *h* = 1 mm, *l* = 2 mm as an example, the plastic deformation behavior of the workpiece edge and the formation process of the exit burr are analyzed according to the simulation results, thereby revealing the suppression mechanism of IBC on the exit burr.

**Figure 5:**
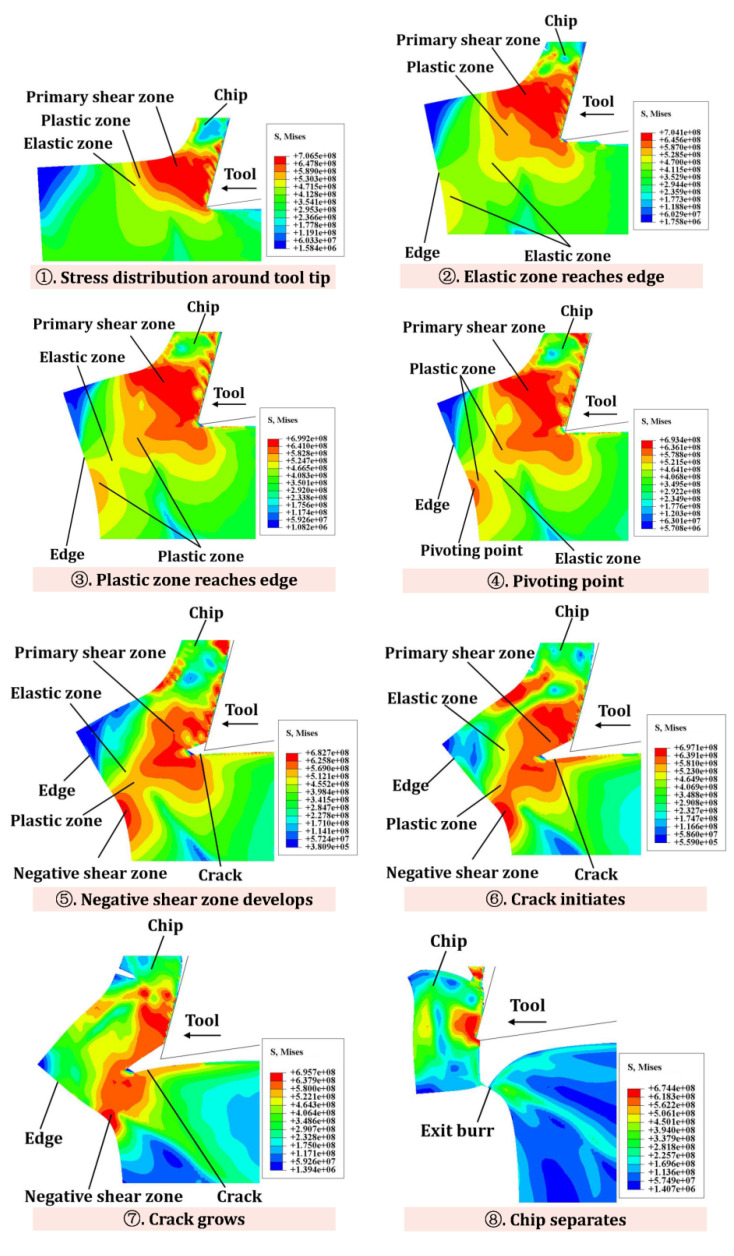
Schematic diagram of stress flow at the workpiece edge.

Figure 5 shows the stress distribution and flow state at the edge of the workpiece without IBC. Stage 1 describes the flow of chip and the state of stress distribution around tool tip during cutting. In stage 2, the elastic zone intersects with the edge of the workpiece, which appears at the workpiece edge as elastic bending. At the same time, the plastic zone around the primary shear zone also tends to move toward the edge of the workpiece. Stage 3 can be seen as the initiation of burrs. At this stage, the obvious plastic deformation occurs at the edges of the workpiece, which is manifested as plastic bending. Additionally, the primary shear zone and the plastic deformation zone around the primary shear zone have a tendency to move towards the edge of the workpiece. In stage 4, a pivoting point where large deformation is clearly visible can be observed at the edge. Stage 5 is the growth of the burrs. In this stage, large plastic deformations of the workpiece edges called the negative shear zone continue to extend and connect with the primary shear zone. As the tool move toward the workpiece edge, the workpiece corner continues to pivot with the cutting chip, so the size of the burr gradually increases. In stage 6, the crack initiates at the tool tip in the primary shear zone. This phenomenon occurs mainly because the material around the tool tip produces a large critical fracture strain. In stage 7, as the tool moves toward the edge, the crack continues to grow along the cutting line, and the workpiece is deformed. Stage 8 shows that the crack causes separation of the chip from the workpiece materials along the cutting line, and the burr remains on the edge of the workpiece. Stages 1–5 explain the formation process of the burrs, while stages 6–8 describe the separation of the chips from the workpiece material as the crack grows.

**Figure 6:**
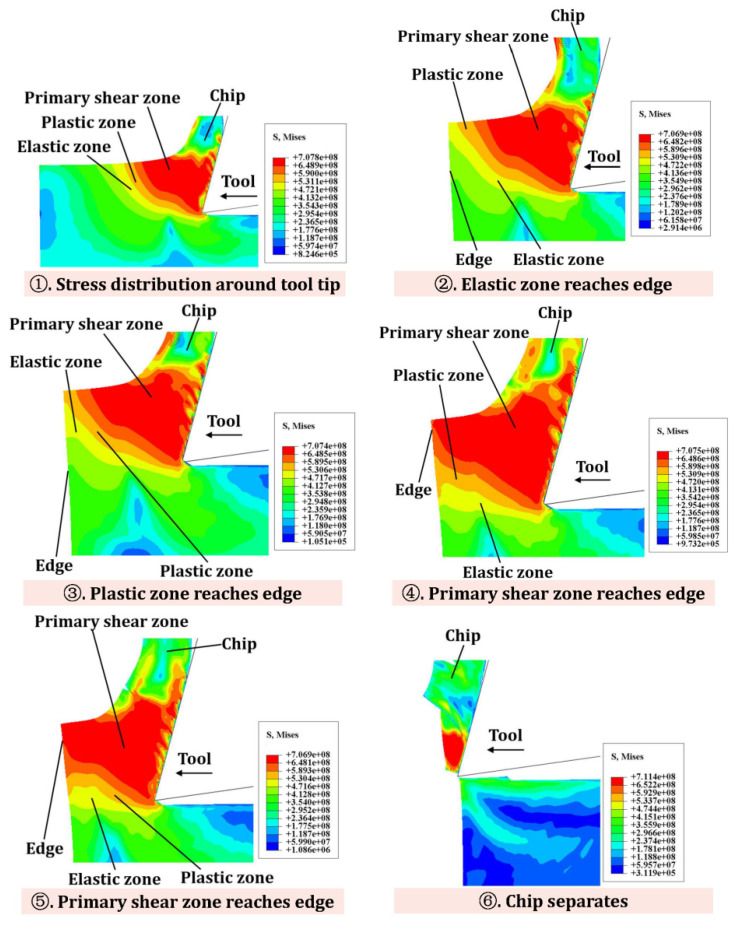
Schematic diagram of stress flow at workpiece edge under IBC.

Figure 6 shows the stress distribution and flow state at the edge of the workpiece with IBC. Stage 1 shows the state of stress distribution of the material around the tool tip. In stages 2–3, the elastic zone and the plastic zone reach the edge of the workpiece, respectively. Stage 4 describes that the primary shear zone around the tool tip gradually moves to the edge of the workpiece, and the workpiece material is slightly deformed. Stage 5 is the process in which the primary shear zone extends along the edge of the workpiece. In stage 6, the chips are separated from the workpiece material, and exit burrs are significantly suppressed.

By comparing the stress distribution at the workpiece edge with/without IBC, the following differences can be drawn (Table 7): (i) As the tool moves towards the workpiece edge, the workpiece edge without IBC will generate a plastic deformation zone and negative shear zone, while the workpiece edge with IBC will not generate a plastic deformation zone and negative shear zone. (ii) Without IBC, the cutting force will cause obvious bending deformation of the workpiece edge. However, the edge deformation under IBC can be greatly suppressed. (iii) Without IBC, the primary shear zone around the tool tip will produce obvious cracks along the cutting line, and the cracks will continue to extend as the tool moves to the edge. However, IBC can suppress the generation of cracks.

## 5. Experimental Verification

**Figure 7:**
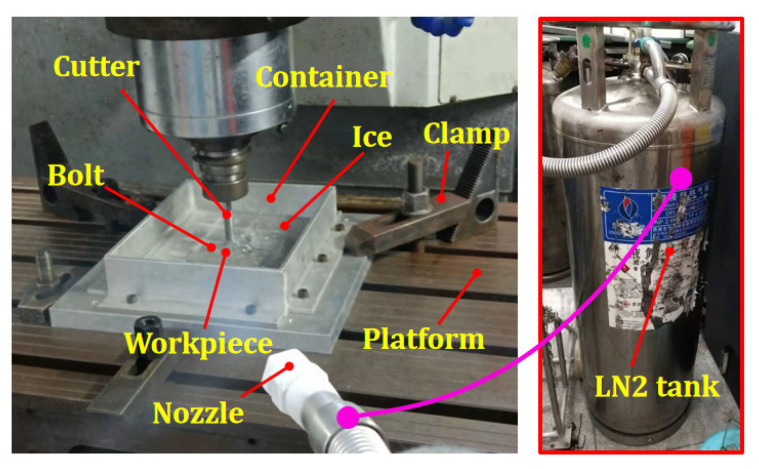
Experiment setups.

The experimental verification was carried out on the KVC850M machine. The experimental setup mainly includes a container, bolts, clamps, a refrigerator, nozzle and LN2 tank (Figure 7). Among them, the bolts are to fix the workpiece in the container. The container is used to hold water. Clamps are used to fix the container to the machine platform. The refrigerator is for freezing water. The nozzle and LN2 tank form a cooling system to cool the machining process.

The experimental steps are as follows: Firstly, the workpiece is bolted to the bottom of the container. Secondly, the container is filled with water, and the workpiece is submerged in water. Next, the container is placed in the refrigerator to freeze. After freezing, the container is taken out from the refrigerator and fixed on the machine platform. Finally, the milling experiment is carried out, and LN2 is sprayed on the machining zone to prevent the ice from melting due to the cutting heat. Cutting parameters and tool parameters are listed in Table 2 and Table 8.

**Figure 8:**
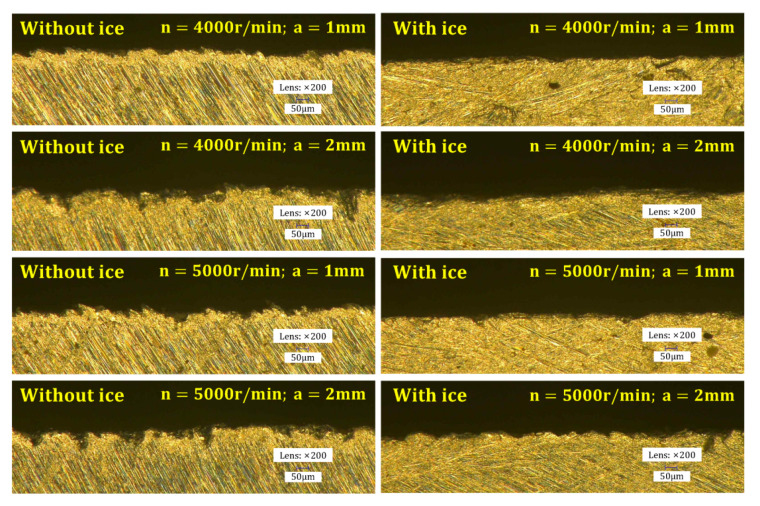
Workpiece edge topography.

**Figure 9:**
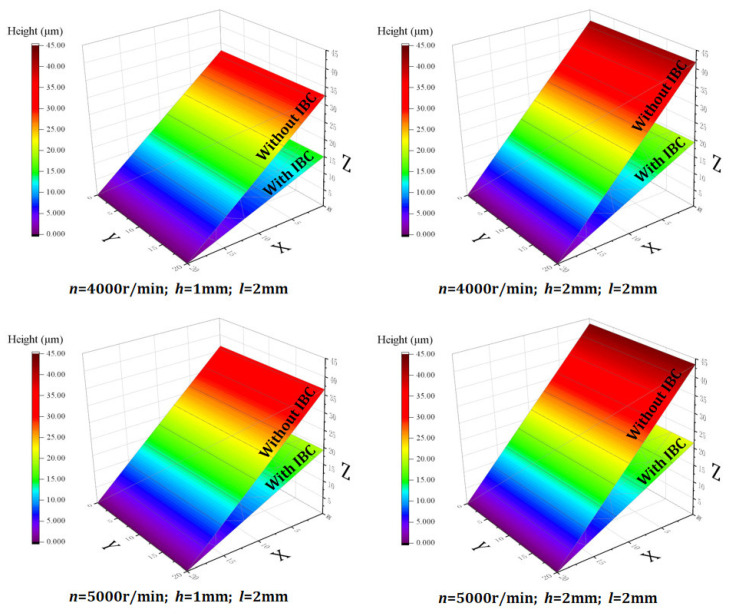
Comparison of average height of exit burr.

The workpiece edge topography was measured by a Keyence ultra-depth three-dimensional microscope. The measurement results show that compared with conventional machining, the IBC method can effectively improve the edge quality of the workpiece (Figure 8) and reduce the exit burr height (Figure 9). Moreover, the quality of workpiece edge changes significantly with the change in cutting parameters under conventional machining, while the quality of workpiece edge is less affected by the change in cutting parameters under IBC.

Although the exit burr is greatly suppressed, it still exists. The main reasons are as follows: (i) the freezing temperature in this study is −20 °C, and at this temperature, the strength of ice is not well matched with the cutting load; (ii) during freezing or machining, ice may produces cracks, which reduces the constraint effect of the ice on the edge of the part; (iii) due to the presence of air in water, tiny air bubbles will be generated at the ice–workpiece interface after freezing, thereby reducing the constraint effect of ice on the edge of the workpiece.

**Figure 10:**
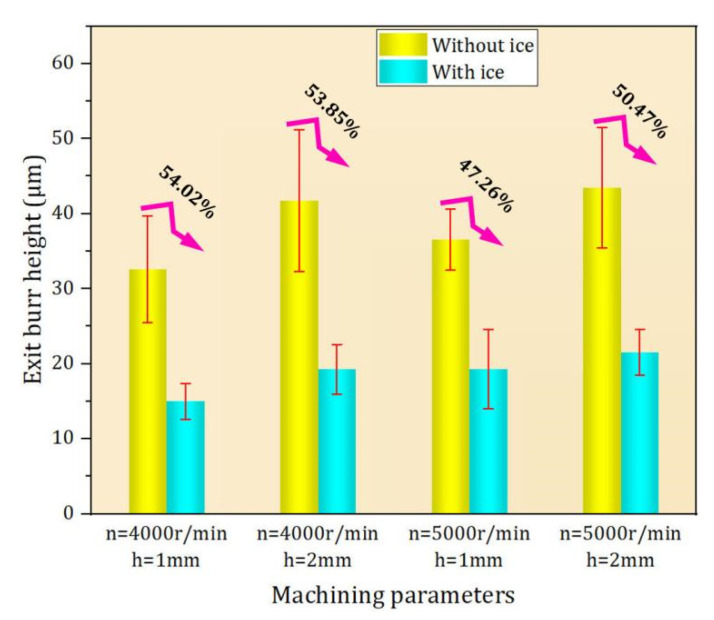
Suppression effect of IBC on exit burr.

**Figure 11:**
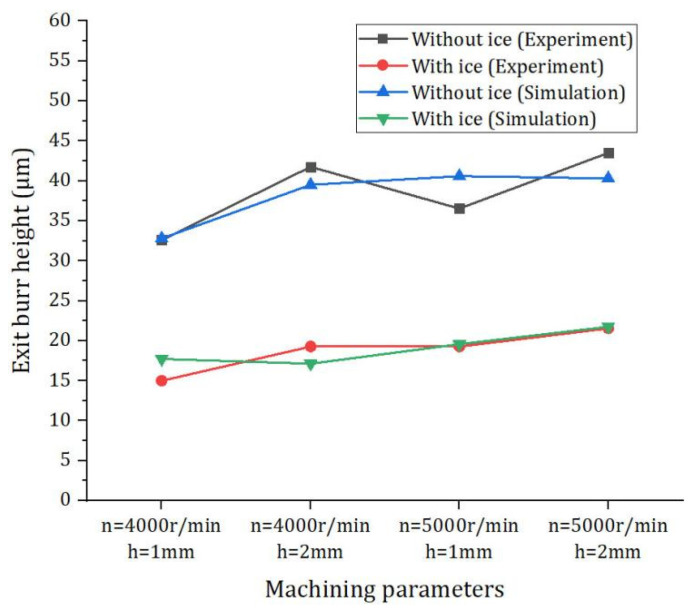
Comparison of simulation results with experimental results.

As shown in Figure 10, when the spindle speed is 4000 r/min and radial depth of cut is 1 mm and 2 mm, IBC can reduce the exit burr height by 54.02% and 53.85%, respectively, and when the spindle speed is 5000 r/min and radial depth of cut is 1 mm and 2 mm, IBC method can reduce the burr height by 47.26% and 50.47%, respectively. In the experiment, the height of exit burr is reduced by 51.4% on average. The experimental results show that the IBC method can effectively suppress the formation of exit burr in milling. In addition, by comparing the simulation results with the experimental results, it can be seen that the finite element model can effectively predict the height of the exit burr (Figure 11). In the experiments, LN2 (−196 °C) was used to cool the machining zone. However, due to the influence of the environment and cutting heat, the temperature of the machining zone is constantly changing. By comparing the simulation results with the experimental results, it can be seen that the friction coefficient in the experiments is close to the friction coefficient of the finite element simulation (μ = 0.2).

## 6. Conclusions

In this paper, a new IBC method is proposed to suppress exit burr formation. Firstly, the formation mechanism of the exit burr is analyzed from the perspective of material flow. Then, the working principle of IBC is explained. Next, FEM is used to analyze the stress distribution and flow state of the workpiece edge under IBC, thereby revealing the suppression mechanism of IBC on exit burr. Finally, the milling experiments are carried out to verify the effectiveness of IBC method and the prediction accuracy of the FEM. The following conclusions can be drawn from this study:

(1) According to the simulation results, it can be seen that IBC can effectively suppress the flow of material caused by cutting stress and reduce the plastic bending deformation at the edge of the workpiece, thereby avoiding the formation of negative shear zone and, ultimately, suppressing the generation of exit burrs.
(2) Under conventional machining, the edge topography of the workpiece changes significantly with the change in cutting parameters. However, the suppressing effect of IBC on exit burrs is less affected by cutting parameters.
(3) The experimental results show that IBC can effectively suppress the formation of exit burrs, thereby improving the edge quality of the part. The comparison results show that the height of exit burr is reduced by 51.4% on average.
(4) The finite element model is verified by the milling experiments. By comparing the simulation results with the experimental results, it can be seen that the finite element model can effectively predict exit burr height in milling experiments.

The author’s research group has applied the IBC method to the processing of some specific workpieces. For example, deformation suppression of cantilever thin-walled parts, suppression of top burrs, precision machining of honeycomb parts, etc. The IBC method can reduce the plastic deformation and cutting heat during the machining process, thereby ensuring the machining quality of the parts. In addition, ice can greatly reduce the temperature of the cutting area, thereby reducing the use of coolant and avoiding the pollution of coolant to the environment. The research in this paper provides some reference and guidance for the precision and clean processing of metal parts in the future.

## Figures and Tables

**Table 1:** Physical properties of workpiece, tool materials and ice.

Properties	AA2024	Carbide Tool	Ice
Density (kg/m^3^)	2700	7900	917
Elastic modulus (Gpa)	73	640	6.05
Poisson’s ratio	0.33	0.22	0.35
Specific heat (J/kg K)	0.557*T* + 877.6	220	2100
Thermal conductivity (W/m K)	0.247*T* + 114.4 (25 < *T* < 300)	75.4	2

**Table 2:** The geometric parameters of the tool.

	Blade Number	Rake Angle	Tool Clearance	Blade Angle	Cutting-Edge Radius	Tool Coating
Tool	3	12°	14°	62°	0.2 mm	No

**Table 3:** J-C constitutive constants for AA2024.

	A (Mpa)	B (Mpa)	C	n	m
AA2024	352	440	0.0083	0.42	1

**Table 4:** J-C damage constants for AA2024.

	*D*_1_	*D*_2_	*D*_3_	*D*_4_	*D*_5_
AA2024	0.13	0.13	−1.5	0.011	0

**Table 5:** The value of the constant.

	$a_{0}$	$a_{1}$	$a_{2}$
Ice	22.39	2.06	−0.023

**Table 6:** Boundary conditions.

Workpiece	Length	Width	High	Materials
	8	4	4	AA2024
Ice	Length	Width	High	Initial temperature
	8	11	4	−20 °C
Process	Radial depth of cut/h	Axial depth of cut/*l*	Spindle speed/*n*	Tool materials
	1 mm, 2 mm	2 mm	4000 r/min 5000 r/min	Tungsten steel carbide

**Table 7:** Analysis of the state of stress distribution on the edge of the workpiece with/without IBC.

	Without IBC	With IBC
Difference 1	As the tool moves to the edge, the edge of the workpiece produces the plastic deformation zone and the primary shear zone in sequence.	As the cutting progresses, no plastic deformation zone and negative shear zone are generated at the edge of the workpiece.
Difference 2	There is obvious bending deformation at the edge of the workpiece.	Only slight deformations occur at the edge of the workpiece.
Difference 3	In the primary shear zone, a clear crack is produced along the cutting line.	No crack is generated in the primary shear zone.

**Table 8:** Experimental design.

No	Spindle Speed (n) (r/min)	Radial Depth of Cut (*h*) (mm)	Axial Depth of Cut (*l*) (mm)	Condition
1	4000	1	2	Without IBC
2	4000	1	2	With IBC
3	4000	2	2	Without IBC
4	4000	2	2	With IBC
5	5000	1	2	Without IBC
6	5000	1	2	With IBC
7	5000	2	2	Without IBC
8	5000	2	2	With IBC

## Data Availability

Not applicable.

## Nomenclature

IBC	Ice boundary constraint	$\delta_{ij}$	Kronecker symbol
FEM	Finite element model	$\mu_{i},\;\lambda_{i}$	Lame coefficient
AA2024	Aluminum alloy 2024	$\varepsilon_{eq}^{p}$	Equivalent plastic strain
J-C	Johnson–cook	p	Hydrostatic stress
σ	Flow stress	$\varepsilon_{f}$	Failure strain
ε	Plastic strain	$\varepsilon_{0}$	Initial failure strain
$\dot{\varepsilon }$	Plastic strain rate	LN2	Liquid nitrogen
$\dot{\varepsilon_{0}}$	Equivalent plastic strain	μ	Friction coefficient
T	Material tempurature	$\tau_{f}$	Friction between the tool and the machined surface
$T_{r}$	Room tempurature	$\sigma_{n}$	Normal stress
$T_{m}$	Melting tempurature	$q_{h}$	Heat dissipation density
A, B, C, n, m	Johnson–Cook constitutive constants	$T_{f}$	Tool temperature
$\sum {\bar{\varepsilon }}^{pl}$	Increment of the equivalent plastic strain	$\bar{h}$	Average convective heat transfer coefficient
$\sum {\bar{\varepsilon }}_{f}^{pl}$	Equivalent strain of fracture	$L_{e}$	Effective cooling strength
$D_{1},\;D_{2},\;D_{3},\;D_{4},\;D_{5}$	Johnson–Cook damage constants for Aluminum alloy 2024	$K_{air}$	Heat transfer coefficient of air
$a_{0},\;a_{1},\;a_{2}$	Ice constants	$P_{r}$	Prandtl number
p	Hydrostatic stress	$R_{e}$	Reynolds number
q	Octahedral shear stress	${\bar{h}}_{e}$	Actual heat transfer coefficient
$d\sigma_{ij}$	Stress increment	λ	Compensation coefficient
